# Evaluation of the Families SHARE workbook: an educational tool outlining disease risk and healthy guidelines to reduce risk of heart disease, diabetes, breast cancer and colorectal cancer

**DOI:** 10.1186/s12889-015-2483-x

**Published:** 2015-11-13

**Authors:** Laura M. Koehly, Bronwyn A. Morris, Kaley Skapinsky, Andrea Goergen, Amanda Ludden

**Affiliations:** grid.280128.10000000122339230Social and Behavioral Research Branch, National Human Genome Research Institute, National Institutes of Health, Building 31, Rm B1B54, Bethesda, MD 20892-2073 USA

**Keywords:** Family health history, Disease risk, Chronic diseases, Family genomics health educator, Risk assessment, Breast cancer, Colorectal cancer, Diabetes, Heart disease

## Abstract

**Background:**

Common diseases such as heart disease, diabetes, and cancer are etiologically complex with multiple risk factors (e.g., environment, genetic, lifestyle). These risk factors tend to cluster in families, making families an important social context for intervention and lifestyle-focused disease prevention. The Families Sharing Health Assessment and Risk Evaluation (SHARE) workbook was designed as an educational tool outlining family health history based risk of heart disease, type 2 diabetes, breast cancer, and colorectal cancer. The current paper describes the steps taken to develop and evaluate the workbook employing a user-centered design approach.

**Methods:**

The workbook was developed in four steps, culminating in an evaluation focusing on understanding and usability of the tool. The evaluation was based on two Phases of data collected from a sample of mothers of young children in the Washington, D.C., area. A baseline assessment and follow-up approximately two weeks after receipt of the workbook were conducted, as well as focus groups with participants. The design of the workbook was refined in response to participant feedback from the first evaluation Phase and subsequently re-evaluated with a new sample.

**Results:**

After incorporating user-based feedback and revising the workbook, Phase 2 evaluation results indicated that understanding of the workbook components improved for all sections (from 6.26 to 6.81 on a 7-point scale). In addition, 100 % of users were able to use the algorithm to assess their disease risk and over 60 % used the algorithm to assess family members’ disease risk. At follow-up, confidence to increase fruit, vegetable and fiber intake improved significantly, as well.

**Conclusions:**

The Families SHARE workbook was developed and evaluated resulting in a family health history tool that is both understandable and usable by key stakeholders. This educational tool will be used in intervention studies assessing the effectiveness of family genomics health educators who use the Families SHARE workbook to disseminate family risk information and encourage risk reducing behaviors.

**Trial registration:**

ClinicalTrials.gov, NCT01498276. Registered 21 December 2011

**Electronic supplementary material:**

The online version of this article (doi:10.1186/s12889-015-2483-x) contains supplementary material, which is available to authorized users.

## Background

Family health history (FHH) is a genomic tool, capturing disease prevalence related to genetic, environmental, and behavioral risk factors that cluster in families [1]. Increased disease risk due to FHH informs clinical care, with those at increased risk recommended to screen more frequently, often at an earlier age [2]. The literature suggests that providing a risk assessment based on FHH is an effective means of educating the public about their disease risk, leading to appropriately adjusted risk perceptions [3, 4] and motivating increases in physical activity and fruit and vegetable consumption [5, 6]. Moreover, those individuals with moderate to high increased risk based on their FHH report increased willingness to speak with their healthcare providers regarding their family risk [7, 8].

While there are online and paper-based FHH tools available, these largely facilitate FHH collection [9, 10] or individualized risk assessment [11] with a focus on easing the FHH collection burden in the clinical setting. Many are designed specifically for use in research or primary care settings, not for public use [12, 13]. Despite the promise of FHH tools for improving health, their utility can be suboptimal if individuals have limited knowledge of their family’s disease history. Indeed, a large portion of the population is missing important FHH information that can impact their health [14].

FHH knowledge is a function of family communication in which family members update each other of disease diagnoses [15]. Consequently, family-based approaches to collect and disseminate accurate FHH information may be a first step in an effective FHH-informed intervention. Women and parents have been shown to play a central role in gathering and disseminating health information to family members, and as such, mothers may be particularly effective as genomics health educators within the broader family system [16–20]. Yet, those who take on this task of gathering and disseminating family risk information commonly express a need for tools to facilitate such conversations [21, 22].

Taking this into consideration, the current project aimed to develop and evaluate a plain language workbook outlining FHH and risk assessment of heart disease, type 2 diabetes, breast cancer, and colorectal cancer. This initial evaluation was conducted with mothers, based on the mounting evidence that women and parents are often the kin keepers of family health information. Given that genomic risk clusters in families, we envision this tool being used by family genomics health educators to disseminate FHH information and associated risk information to relatives and encourage attainable screening and health promoting lifestyle behaviors. This family genomics health educator model is grounded within the communal coping framework, which is characterized by three interpersonal processes: 1) communication about a shared health threat, for example, inherited disease risk; 2) formation of shared appraisals of the threat; and 3) initiation of cooperative strategies to reduce the threat [23, 24]. As such, FHH-based risk information can motivate communal coping processes, with the goal of activating cooperative strategies that shift familial norms around healthful, risk reducing behaviors. Theoretically, such healthful familial norms have important implications in sustainable engagement in preventive health behaviors [25–27].

**Fig. 1:**
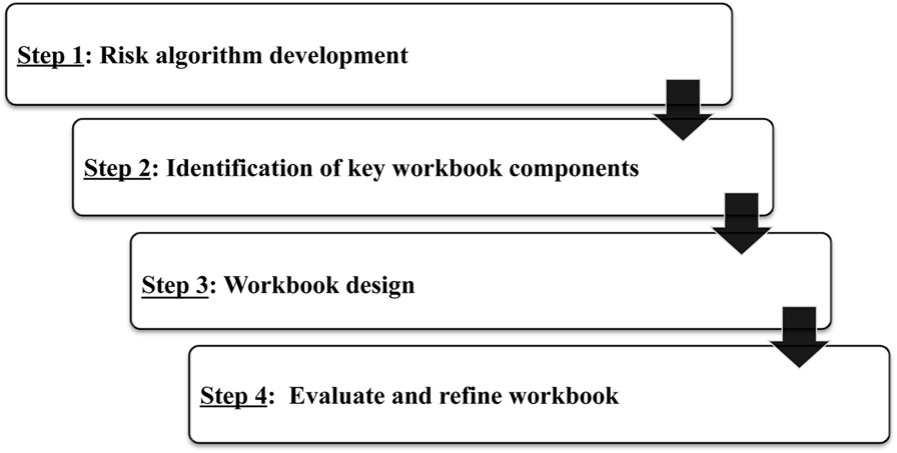
Schema used to develop and evaluate the Families SHARE workbook. The Families SHARE workbook was developed and evaluated within a four step process. In the first step, we developed the family health history based risk algorithm based on a systematic review of the literature. Step 2 involved identification of key workbook components using concepts from the Health Belief Model; these components were integrated into the workbook design in Step 3. A rigorous evaluation was completed in Step 4. The evaluation was conducted in two Phases such that the workbook was revised based on recommendations from Phase 1 and re-evaluated in Phase 2

The current report focuses on the development of an acceptable, understandable, and usable tool to facilitate communication of FHH information. The purpose of the Families Sharing Health Assessment and Risk Evaluation (SHARE) workbook is twofold: first, to engage active learning processes and educate individuals regarding their family risk of disease by connecting their FHH to risk through a simplified risk algorithm; and second, to provide behavioral and screening guidelines that have the potential to reduce disease risk. By providing a risk algorithm, the user becomes actively engaged in computing her own as well as her family members’ FHH-based risk; such active participation is hypothesized to facilitate an individual’s understanding of FHH and its relevance to health both for the user and their broader family system. Through an understanding of family members’ shared risk, interpersonal mechanisms that shift family norms towards healthful, risk-reducing behaviors may be activated. The workbook development and evaluation process, outlined in the current report, was completed in four steps which are detailed in Fig. 1 and the Methods section that follows.

## Methods

### Step 1. Development of risk algorithms for heart disease, type 2 diabetes, breast cancer, and colon cancer

The first step in creating the Families SHARE workbook was to develop an accurate, yet simple, algorithm for assessing increased common disease risk due to FHH. The four diseases included in the workbook are among the 15 leading causes of death in the United States [28] and were selected for having both familial and modifiable behavioral risk factors. While there are a multitude of complicated algorithms used to assess disease risk [29–31], these algorithms may not be easily implemented within a community-based public health intervention and interpreted by participants [32]. Thus, this first step focused only on FHH based risk, constructed from a review of the literature and practitioner guidelines for stratifying screening recommendations in clinical practice.

A preliminary literature search yielded numerous FHH-inclusive risk assessment models for heart disease, type 2 diabetes, breast cancer, and colorectal cancer. The language and statistics used to communicate disease risk varied across these models, as did the criteria for risk assessments bearing the same label. For example, Snyder et al. ascribe a “high risk” of breast cancer to individuals with at least one first-degree relative (FDR) diagnosed under the age of 50 [33], while Warner et al. consider the same FHH to warrant only “moderate risk” [34]. Other models eschew verbal descriptions and instead convey risk in odds ratios or risk points, generally referring to an increased risk [35].

**Fig. 2:**
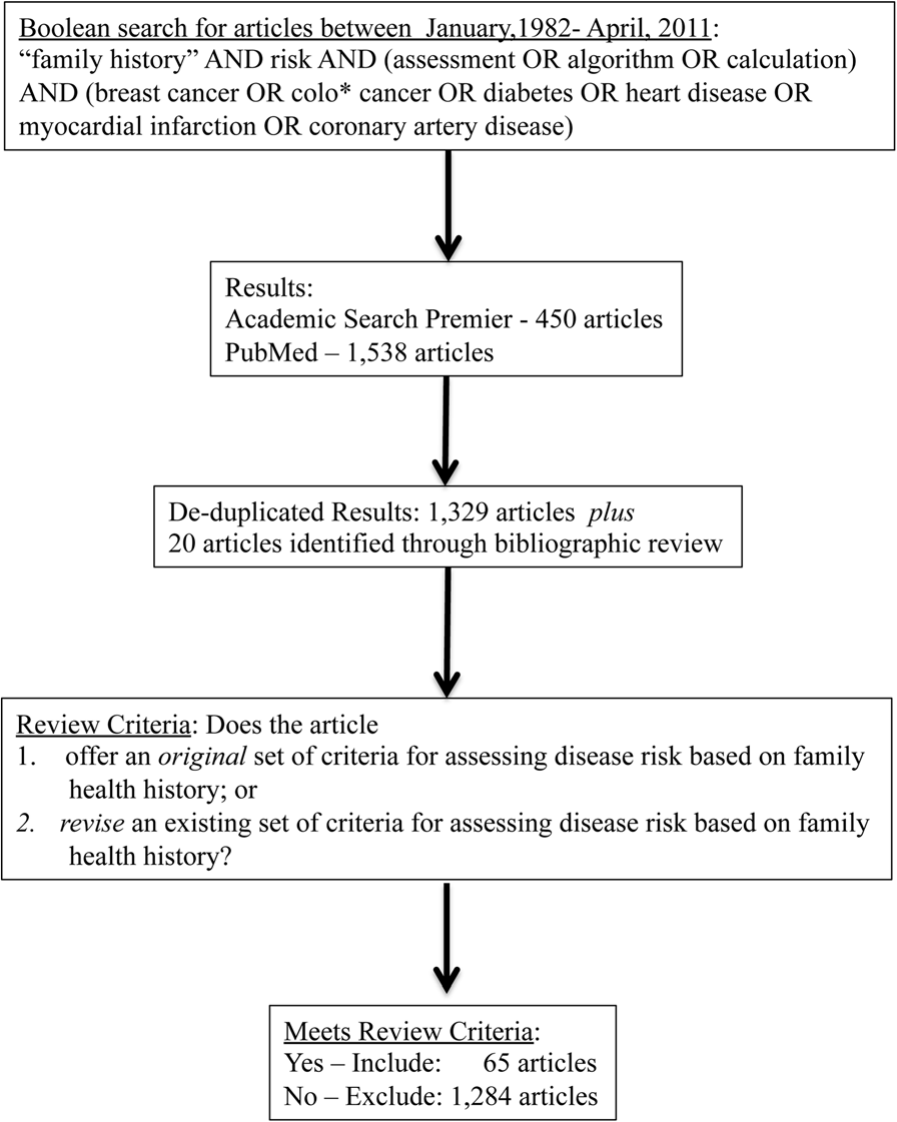
Flow diagram for systematic review of the literature on family health history based risk assessments. This figure characterizes the process guiding the systematic review of the literature on family health history-based disease risk algorithms. The first step involved searching within Academic Search Premier and Pub Med based on a set of key search terms, limiting years of publication between January, 1982 and April, 2011

Thus, it was decided that the risk algorithm developed for the Families SHARE workbook should incorporate the “least common denominator” of risk criteria while being stringent enough to preclude overestimation of risk. To determine these criteria, a systematic literature search was conducted throughout the Academic Search Premier and PubMed databases. The terms “‘family history’ and risk and (assessment or algorithm or calculation) and (breast cancer or colo* cancer or diabetes or heart disease or myocardial infarction or coronary artery disease)” yielded 1,329 results published between January, 1982 and April, 2011 (see Fig. 2).

A manual search was then conducted through articles’ bibliographies to account for sources not captured by the original search terms, yielding another 20 articles for review. In total, 65 articles were included for review that 1) offered an original set of criteria for assessing disease risk based on FHH only or controlling for demographic characteristics; or 2) revised an existing set of criteria for assessing disease risk based on FHH only or controlling for demographic characteristics. Articles in which family history contributed points towards a total risk score were excluded from the review [35–41]. Similarly, risk assessments that considered FHH in a multivariate model including clinical or behavioral risk factors (e.g. BMI, breast density, biomarkers, or lifestyle factors) were not synthesized within the review. Twenty three of the 65 articles featured categorical risk language, while the remainder focused on continuous risk variables (e.g., odds ratios, relative risks, hazard ratios) [42–84]. Risk criteria and risk assessment language were cataloged from these articles, and similar terms were then rephrased to facilitate grouping, e.g. “one FDR relative with CRC” [85] and “family history of colorectal cancer: any FDR” became “affected FDR” [77], a factor associated with various categorical expressions including “high risk,” “increased risk,” and “moderate risk,” depending on the source paper (Additional file 1).

The risk criteria and risk assessment language indicated an increased risk based on the same criteria for each of the four diseases: subjects are ascribed “increased risk” for any of the four diseases if they have at least one affected FDR or two affected second-degree relatives (SDRs) with a specific disease. This “increased risk” category generally corresponds to ratios or relative risks at or above 2.0 [42, 44, 45, 47–49, 52, 53, 55, 57, 59, 60, 63–65, 68–70, 72–74, 76, 77, 79, 83]. Thus, Families SHARE adopted this risk algorithm based on this aggregation of risk criteria and risk language, as well as the recommendations of two genetic counseling experts. This increased risk label is consistent with increased frequency of screening in clinical practice [86–88].

### Step 2. Identification of key factors to be communicated in workbook

In addition to the risk algorithms, information that would facilitate conversation about risk and promote encouragement of risk-reducing strategies was also included [89]. Specifically, as presented in Table 1, workbook content maps to fundamental concepts within the Health Belief Model (HBM) [90]. To improve knowledge regarding family risk of common, complex disease, a personalized three-generation health pedigree was provided with the Families SHARE workbook which includes information on how to interpret key components of the pedigree, along with an example. The risk algorithm provided users with a way to update their perceived susceptibility of disease, with a focus on increased risk versus population-based risk. Each disease is defined and a list of behavioral risk factors is provided. Finally, to encourage positive health behaviors, the workbook presented information intended to increase the perceived benefits of and cueing behavioral actions aimed at disease prevention or early detection. The workbook highlighted demonstrated, modifiable risk-reducing strategies that have been identified as leading causes of death in the United States – namely, smoking behavior, poor diet, physical inactivity, and alcohol use [91]. Such factors have been shown to be associated with the focal diseases presented in the Families SHARE workbook [2, 92–96].

**Table 1:** Health belief model concepts mapped to workbook components

Concept	Definition	Component
Perceived susceptibility	Beliefs about the chances of getting a condition	• Family health history • Personalized risk assessment based on FHH for self • Personalized risk assessment based on FHH for relatives
Perceived severity	Beliefs of how serious a condition is	• Define the disease • Provide information regarding risk factors for a disease
Perceived benefits	Beliefs about the effectiveness of advised actions to reduce risk of the condition	• Screening recommendations, with frequency based on risk • Lifestyle recommendations, with suggested approaches for implementation in • daily life
Cues to action	Strategies for initiating actions for risk reduction	• Recommendations to share FHH risk information with health care providers • Recommendations to share FHH with family members • Worksheets for risk assessment for family members

### Step 3. Design materials that emphasize key concepts chosen for communication

Messaging was developed based on a review of the literature, government resources (e.g CDC, NIH, AHRQ), and expert websites (e.g., Mayo Clinic). All content was presented at a Grade 8 reading level (Flesch-Kincaid). The first version of the workbook was sent to experts in health literacy and genetics education for review and comment. Following initial revision, materials were distributed to a sample of key stakeholders (mothers of young children) from the community for evaluation.

### Step 4. Evaluate and refine materials using input from stakeholders

The workbook materials were further refined based on the responses from key stakeholders in two Phases. This evaluation was approved by the Institutional Review Board of the National Human Genome Research Institute (NHGRI: 12-HG-0023) and registered in ClinicalTrials.gov (NCT01498276). The procedures and results for these two Phases of evaluation are detailed below.

## Participants and procedures

**Fig. 3:**
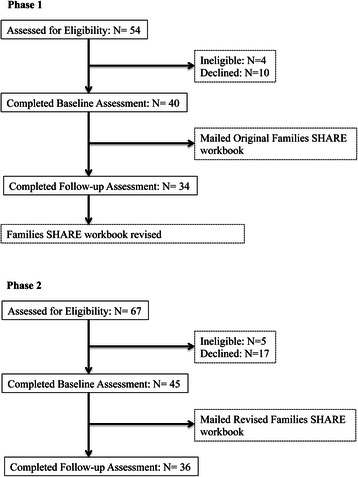
CONSORT diagrams for the Phase 1 and Phase 2 evaluation of the Families SHARE workbook. The evaluation of the Families SHARE workbook was conducted in two Phases. In Phase 1, 40 mothers were successfully enrolled from the 50 eligible persons contacted; 34 (85 %) completed follow-up assessments. The workbook was revised based on user recommendations through interview and focus groups. In Phase 2, the revised Families SHARE workbook was evaluated within a new sample. Forty-five mothers were successfully enrolled from the 62 eligible persons contacted; 36 (80 %) completed follow-up assessments

Participants were recruited from an existing database of mothers (≥18 years of age) with young children who had agreed to be contacted for future studies. The CONSORT diagram for both Phases of the evaluation is provided in Fig. 3. Phase 1 of the evaluation recruited 40 participants, who gave verbal informed consent and completed surveys via telephone interviews. Participants were mailed a Families SHARE workbook, including a personalized pedigree, after completing the baseline survey, which collected a detailed FHH for heart disease, type 2 diabetes, breast cancer and colon cancer. Participants were contacted for a follow-up telephone survey to evaluate their understanding of the materials on average 2 weeks after sending the workbook. A total of 34 participants completed both baseline and follow-up assessments. A subset of participants also attended focus group sessions after completion of the follow-up survey to elaborate on issues assessed in the survey. Following Phase 1, the workbook was revised based on participant feedback and presented to an additional 45 participants recruited for Phase 2 of data collection. Initial and follow-up surveys were also completed via telephone interviews following verbal informed consent in Phase 2, and a third focus group was conducted to evaluate the revised workbook. A total of 36 participants completed both assessments in Phase 2.

Focus group sessions were approximately 1.5 h and were conducted by a trained moderator and note taker. Semi-structured topics guided the focus group sessions in order to assess participants’ understanding of and use of the workbook. Focus groups followed the semi-structured topic guides (see Table 2), using open-ended questions and prompts to explore each topic. Focus groups were recorded and transcribed.

**Table 2:** Focus group semi-structured topic guide

Understanding of and assessment of utility of the Families SHARE workbook	
1.	How useful was the workbook in helping you understand your risk of disease?
2.	Out of the three steps of the workbook (the sample exercise, your own FHH tree, and the health guidelines):
	• Were there any steps that stood out as being the most helpful? • Were there any steps that were confusing?
3.	Does the sample exercise increase your understanding of your own Family Health History?
4.	What could we add or improve to make the workbook better and help you understand you or your family’s disease risk or ways to reduce your risk?
Communication and encouragement	
1.	When you were filling out the initial survey for us with the family history details, did you talk with anyone to get health information? Who did you talk with?
2.	Did you show the workbook to anyone else in your family or to others outside of your family? How did you do that? (e.g. sit down with them and “teach” them how to evaluate their risk or just give them the workbook?)
3.	Do you plan on showing the workbook to anyone else? (e.g., health care providers)
4.	Did you and your family discuss ways to reduce your disease risk? Is this important to you and/or your family?

## Measures

### Demographics

Demographic characteristics, including age, marital status, race/ethnicity, household income and education were assessed at baseline.

### Health behaviors

Self-reported height and weight were assessed at baseline and converted into Body Mass Index (BMI). Additionally, self-reported lifestyle behaviors were assessed at baseline, including vigorous and moderate physical activity, cups of fruit and vegetables eaten each day, and smoking behavior [97, 98]. These items were converted to assess the percentage of the sample meeting Centers for Disease Control and Prevention (CDC) recommendations [99].

### Recommendations for improvement

In a free response format, respondents indicated elements of the workbook that were confusing and offered suggestions for improvement.

### Understanding of workbook

At follow-up, participants completed a series of questions assessing workbook usability and understanding. Usability questions asked whether participants were able to use the algorithms for themselves and other family members. Understanding questions asked participants to indicate on a 7-point scale their understanding of the workbook overall and their understanding of individual components of the workbook (e.g. sample pedigree, personal pedigree, disease risk worksheets, etc.), with 1 representing “I did not understand at all”, three representing “I understand a little”, five representing “I mostly understood”, and seven representing “I completely understood.” A 7-point scale was used to maximize variability and reliability in responses [100]; in addition, intermediary responses were provided to ease translation of response options.

### Intention and confidence to change behavior

At baseline and follow-up, participants were asked to indicate: “From the following list, are there any lifestyle behaviors that you would like to improve?” The list included: limit alcohol consumption, stop smoking, increase fruit and vegetable consumption, increase fiber consumption, increase physical activity, none of the above, other – free response. In this report, we focus on dietary behaviors and physical activity; a dichotomous variable was created indicating whether the participant intended to change the selected behavior. For each indicated behavior, participants were asked to rate their confidence in making changes within the next year on a 7-point scale, where one is “not at all confident” and seven is “very confident.” These items were mapped to the lifestyle messages presented in the workbook; a 7-point scale was used to maximize variability and reliability based on guidelines put forth by Krosnick and Presser [100]. The intentions and confidence items were combined into one measure for each behavior, whereby those indicating no intention to change a particular behavior were assigned a confidence score of 0, and others assigned the value selected for their confidence rating.

## Analysis plan

### Survey data

Survey data were analyzed using SPSS and R. Descriptive measures, including means, standard deviations, and ranges, were computed for sample characteristics, shifts in confidence to modify behavior, understanding workbook components, and utilization of the workbook for self and other family members. Where appropriate, paired t-tests were computed to evaluate differences between baseline and follow-up assessments for each Phase; hypothesis testing was conducted using a type 1 error rate of .05. A content analysis was conducted to aggregate free response data.

### Focus group data

Focus group audio recordings were transcribed and investigated using thematic analysis, based in an interpretative phenomenological framework that is extensively used in health psychology research [101]. This framework was utilized to understand the lived experience of the participants and adopts a shared understanding from the perspective of the researcher and the interviewer [102]. As emergent themes were identified, the transcripts were continually reviewed and similar concepts were grouped into superordinate themes [101]. Overarching super-ordinate themes emerged from the identified component themes through an iterative process, and data saturation was reached. Prior theory served as a resource for interpretation of themes, and component and super-ordinate themes were discussed amongst authors and confirmed against the transcript data. Two researchers performed thematic analysis separately using NVivo (version 9.2) and strong inter-rater reliability was found, with substantial Kappa values (0.77) and 97.92 % agreement averaged across the themes.

## Results

### Participant characteristics

Table 3 describes the samples from both Phases. There were no significant differences between the two samples on demographic or health behavior characteristics. The majority was between the ages of 31-40, white, married, had completed post-graduate education, and had annual household income that exceeded $100,000. In terms of health status and associated behaviors, participants were largely healthy. The majority met recommendations for daily moderate physical activity and did not smoke. Approximately half met daily fruit and vegetable intake recommendations.

**Table 3:** Participant characteristics

	Phase 1	Phase 2
	(*n* = 35)	(*n* = 36)
	M (SD)	M (SD)
Age	39.3 (5.0)	37.3 (5.4)
Family size		
Number first degree relatives	6.20 (1.66)	6.14 (1.66)
Number second degree relatives	9.34 (3.13)	11.94 (5.21)
Race	%	%
Black, or African American	20.0	13.9
White	77.1	61.1
Other	2.9	25.1
Marital status		
Single	11.4	16.7
Married	82.9	75.0
Separated/divorced	5.8	8.3
Education		
High school diploma/GED	2.9	2.8
Associate degree/some college	11.4	11.1
Bachelors degree	37.1	22.2
Post-graduate degree	48.6	63.9
Household Income		
Below $50,000	8.6	11.1
$50,001–100,000	17.2	33.3
Greater than $100,000	74.3	52.8
Refuse	–	2.8
Meets daily health recommendations		
Non-smoking	100.0	94.4
Fruit intake (1.5 cups)	58.8	50.0
Vegetable intake (2 cups)	50.0	47.2
Moderate physical activity (≥30 min)	58.8	66.7

*Abbreviations*: *M* mean, *SD* standard deviation

### Phase 1 recommended changes and areas for improvement

Through open-ended questions, as well as feedback from focus groups, we identified several key areas for change based on participant confusion, requests for additional information, or requests for clarity on presented information. These recommended changes, as well as the consequent workbook modifications to address recommendations, are summarized in detail in Table 4.

**Table 4:** Key recommended changes and workbook modifications

Phase 1 recommended changes	N	Workbook modifications
No changes	11	
General recommendations		
Add more information about the motivation for the workbook	4	The introduction page was revised to provide context about the research study and the value of the workbook.
Include disease information fact sheets	3	Disease information fact sheets were added including definitions of each disease and other pertinent information, such as risk factors and health screenings.
Add ideas on how to share risk information with relatives	4	Text was added encouraging use of the workbook to share this information with family members and health care providers, as well as the addition of a study website to access risk evaluation worksheets for other family members, such as children.
Visual improvements (less text, larger print, more graphics)	9	Text size was increased, text amount was decreased, and more graphics were added.
Reorder pages and add space to take notes	7	Two full pages are dedicated to each disease and demarcated with labeled tabs, allowing participants to go directly to the disease most salient to them.
Sample and personal family health history trees		
Remove, shorten, or clarify sample pedigree and sample assessment	14	The revised sample pedigree section was shortened and clarified for use as a reference instead of an exercise. Participant action is now focused on their personal risk assessment worksheets for each disease.
Add color and symbols to represent different diseases	4	No change.
Clarify first and second degree relatives visually	7	First- and second-degree relatives are now defined with text, plus color is used for clarification and better understanding.
Risk assessment		
Use a numerical risk assessment	4	No change.
Make the risk assessment interactive	3	Risk assessment was simplified and made more interactive through the use of worksheets that refer to their personal pedigree.
Behavioral recommendations		
Include concrete recommendations and links to resources	8	Generic information was replaced with more concrete, creative information to make health recommendations more actionable.
Clarify screening recommendations based on risk assessment	3	To connect the family history risk assessment worksheets with health behaviors, relevant behavior and screening messages were added below each risk evaluation.
Add a screening behavior timeline	2	No change.

*Abbreviations*: *N* Number participants recommending this change

The main themes that emerged from focus groups included 1) confusion about the purpose of the workbook, 2) the need for interactive worksheets, 3) definition of risk and becoming aware of risk, 4) reducing generic information, 5) focusing on children’s disease risk and behaviors, 6) aesthetics, and 7) application of workbook.

#### (Theme 1) Confusion about the purpose of the workbook

Participants reported needing more context and background information to guide the user through the packet. “I didn’t totally understand what the goal was of the packet … and I wish that had been sort of, you know, front and center.”

#### (Theme 2) Need for interactive worksheets

Participants reported having to write down information in order to calculate their disease risk. Worksheets built into the workbook were suggested as an interactive method for people to engage in the activity of calculating risk. For example, “I definitely wanted the worksheet that you mentioned [by a participant earlier in focus group], because I felt like I was just writing it on the side and flipping back and forth. So I think that would help. Like one for each of the diseases.” The possibility of an online disease risk calculator was also raised.

#### (Theme 3) Definition of risk and becoming aware of risk

Participants wanted to know what “*increased risk of disease”* meant and how this related to the likelihood of being diagnosed with this disease. They wanted more information about each disease. “I would be interested in having more numbers and, uh, you know having an increased risk it can be a serious thing, but it can be nothing.”

#### (Theme 4) Reducing generic information

Participants indicated that the workbook contained too much generic information. Participants needed assistance to build personal mental models of how health behaviors connect to disease risk, as well as specific tips for becoming healthier. For example, rather than a general statement of “eat fiber,” participants requested specific ways to incorporate the recommended amount of fiber in their diets. “If you say, eat more fruits and vegetables and you don’t tell me why, okay…how does that affect me?”

#### (Theme 5) Focus on children’s disease risk and behaviors

Participants discussed focusing the workbook on their children’s FHH and risk. Women also reported that they are more likely to encourage the rest of their family to be healthy but not look after themselves in the same manner. “I make my daughter eat her vegetables and sometimes I slide them on my plate, like ‘I’m not eating that today.’ So,… I think I care more about my child than I really care about myself, to be honest with you.”

#### (Theme 6) Aesthetics

The pedigree in the workbook needed to be more visually appealing. Overall, the text size throughout the packet was discussed as being too small with too much information crowded on one page. The overall flow of the document needed to transition more smoothly as the ordering of the information was too confusing. For example, “On page two, my initial instinct was that this [sample] is too confusing and way too complicated and I don't have the time to read it.”

#### (Theme 7) Application of workbook

Participants reported the benefit of seeing their FHH written down. For example, “It’s one thing to know if your family's at risk, but when you actually see it written down and out, it’s kind of like it’s in your face. So, it kind of makes you more aware.” Participants discussed the benefits of making the workbook into an online application. However, they also reported being worried about ramifications from insurance companies discovering this information.

### Workbook modifications based on Phase 1 recommended changes

The detailed feedback collected from participants through interviews and focus groups engendered substantial revisions to the workbook between Phase 1 and Phase 2. Here and in Table 4, we highlight some of the key revisions motivated by the insights we synthesized from this feedback. First, the introduction page was thoroughly reworked to provide participants with greater context about the research study and the purpose of the workbook (Theme 1). Participants were also encouraged to use this workbook as a launching point to other critical actions, such as sharing this information with family members and a physician, and visiting the study website to access risk evaluation worksheets for other family members, such as children (Theme 5).

**Fig. 4:**
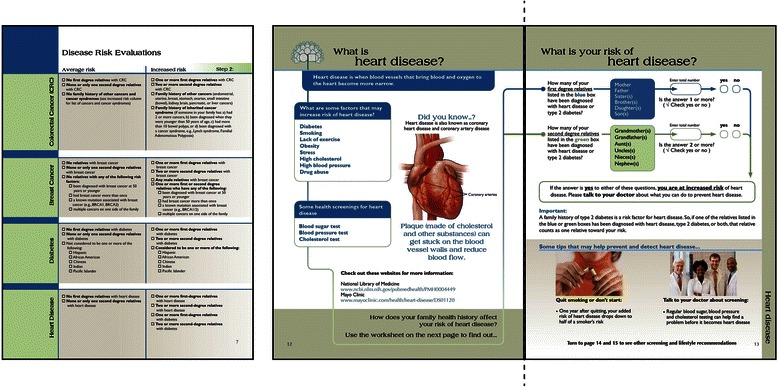
Risk evaluation section in Phase 1 workbook (left) compared to Phase 2 workbook (*right*). These images highlight the major differences between the disease risk evaluation sections in the initial version of the workbook, as compared to the revised version of the workbook. The main revisions include a separate worksheet for each of the four diseases (heart disease shown here); a fact sheet on the left with definitions, risk factors, screening information, and disease specific online resources; a worksheet format for applying the risk algorithm instead of a checkbox system; and disease tailored prevention and screening behavioral recommendations

In the body of the first version of the workbook, participants were provided a sample pedigree populated with hypothetical FHH information, to facilitate understanding of how to navigate and interpret FHH-based risk. In response to participant feedback, the revised sample pedigree exercise is considerably shorter and more straightforward, and delineates first- and second-degree relatives with both text and color to define these essential concepts more clearly (Theme 6). The disease risk evaluations also evolved substantially between Phase 1 and Phase 2 (see Fig. 4). Rather than clustering all four disease risk evaluations on a single page, the revised version of the workbook dedicates two full pages to each disease, allowing participants to go directly to the disease most salient to them. This new organization scheme also afforded space to define each disease clearly and provide disease specific risk factor and health screening information (Theme 3). The process of calculating risk became simpler and more interactive, simply prompting participants to count and record the number of first- and second-degree relatives who have certain diagnoses and directing those participants at increased risk to contact a physician (Theme 2).

To facilitate participants building mental models of how health behaviors influence disease risk, we included a relevant behavior and screening message below each risk evaluation. For the final section of the workbook, dedicated to providing participants with health behavior and screening information, we replaced generic information with more concrete, creative information to make it more actionable and empowering (Theme 4). For example, the revised version of the message to be physically active included information on the impacts of physical activity on the body, such as strengthening bones and muscles and lowering blood pressure.

### Phase 2 recommendations and areas for improvement

Sixty-seven percent of the participants in the Phase 2 evaluation, in contrast to 32 % in Phase 1, had no recommendations for workbook modifications to improve understanding, demonstrating significant improvement based on Phase 1 feedback (*p* = .004). Most recommendations for improvement did not converge on specific changes, but rather represented a single participant’s recommendation. Examples include simplifying the disease fact sheets (*n* = 1), providing more disease facts (*n* = 1), providing more background regarding why the four conditions are the focal point (*n* = 1) and why FHH is important (*n* = 1). Three participants recommended a more quantified risk assessment, with one wanting integration of lifestyle habits as a potential adjustment to the risk assessment. Participants requested more tips on how to implement both lifestyle and screening recommendations, particularly when at increased risk of disease (*n* = 4). Additionally, participants requested more information to facilitate family communication (*n* = 1) and a child version of the packet (*n* = 1).

The main themes emerging from the Phase 2 focus group included: 1) disease interactive worksheets, 2) focus on children’s disease risk and behaviors, and 3) sharing with family.

#### (Theme 1) Disease interactive worksheets

Participants liked the color-coded, disease specific worksheets, with each disease separated by tabs. They agreed on wanting more information on clear actions to take if at increased risk as well as signs and symptoms to look for and how to discuss disease risk with their doctor. However, there was a lack of consensus among participants regarding some of the workbook revisions, including the amount of information provided (some wanted more, some wanted less) and the location of disease information relative to the risk assessment.

#### (Theme 2) Focus on children’s disease risk and behaviors

None of the participants accessed algorithm worksheets for children and other family members that were made available on the Families SHARE website and noted in the workbook introduction. However, all were interested in a shift of focus towards their children’s risk; thus, wanting a more family-oriented workbook, instead of an individually-oriented workbook that incorporated their children’s fathers’ FHH and disease risk. Additionally, assuming a shift in focus towards children’s risk, participants indicated that they wanted more information on what to do if their child was at increased risk. For example, “when your kid hits 25, this is what he should be tested for, or something. I think that would be helpful.” In addition, tips for mothers on how to improve their child’s lifestyle factors, such as a website to go to that has information on “how to get my kid to eat vegetables” or adding “fiber to kids’ meals”.

#### (Theme 3) Sharing with family

In addition, participants expressed a desire to share information with other members of their family and to have an online link. “It would be good if you could share it with the other members of your family. So I could send all my health history that I’ve created to my sisters, ‘cause this is their history too…” “If I could do it online and just send them a link or say ‘here’s an … invitation’ to view the health history… to make changes.”

### Usability and understanding of Families SHARE workbook

Table 5 provides summary statistics related to understanding of the workbook components and engagement in workbook concepts across the two phases. Overall, participants indicated high levels of understanding of workbook components. In Phase 1, lowest levels of understanding were indicated for the sample pedigree (*M* = 5.89, SD = 1.49); following revision, Phase 2 participants indicated increased understanding of the sample pedigree (*M* = 6.61, SD = 0.90). The ranges and standard deviations of responses were much narrower and the means were higher in the Phase 2 evaluation, suggesting improvement in understanding across all workbook components.

**Table 5:** Participant understanding and engagement in the Families SHARE workbook

	Phase 1		Phase 2	
	(*n* = 35)		(*n* = 36)	
Understanding	Min-Max	M(SD)	Min-Max	M(SD)
Overall impressions	3–7	6.03 (1.15)	4–7	6.26 (0.95)
Sample family health history tree	1–7	5.89 (1.49)	3–7	6.61 (0.90)
Personal family health history tree	1–7	6.37 (1.24)	5–7	6.64 (0.59)
Disease risk algorithm	3–7	6.14 (1.03)	5–7	6.44 (0.77)
Health guidelines for risk reduction	4–7	6.76 (0.61)	5–7	6.81 (0.47)
Engagement	%		%	
Able to assess personal risk using algorithm	91.4		100	
Able to assess family members’ risk using algorithm	65.7		61.1	
Talked to family to obtain family health history information	34.3		27.8	
Talked with child’s father to obtain his family history information	31.3^a^		38.9	
Talk with family regarding disease risk	5.7		19.4	

*Abbreviations*: *M* mean, *SD* standard deviation

^a^3 responses missing

The majority indicated that they were able use the risk algorithm to calculate their own risk and the risk of at least one family member during both evaluation Phases. While about a third of participants indicated gathering FHH information from their family or their child’s father during both evaluations, there was a slight reduction in collecting FHH information from family and a slight increase in collecting FHH information from their child’s father for Phase 2 participants. Almost 20 % discussed FHH based disease risk with their family at Phase 2 compared to 6 % during Phase 1, a 3-fold increase.

### Impact of families SHARE workbook on behavioral intention and confidence to change behavior

Table 6 demonstrates a general trend in increased intention and confidence to change behavior between baseline and follow-up assessment for both evaluation Phases. Workbook content appeared to consistently increase intention and confidence to improve dietary behavior. However, intention and confidence to improve physical activity was not affected by the Families SHARE workbook. Given that baseline confidence was highest for physical activity, this null effect for increased intention and confidence to improve physical activity may reflect a ceiling effect.

**Table 6:** Shifts in intention and confidence to modify behavior in next year

	Phase 1			Phase 2		
	(*n* = 35)			(*n* = 36)		
	Baseline M(SD)	Follow-Up M(SD)	*p*-value	Baseline M(SD)	Follow-Up M(SD)	*p*-value
Intention and confidence to change behavior						
Increase fruit and vegetable consumption						
All participants	3.66 (2.51)	4.77 (2.44)	.048	3.33 (2.61)	4.25 (2.61)	.021
Subset with intentions to improve behavior at both assessments^a^	4.83 (1.50)	5.57 (1.16)	.004	4.79 (1.64)	5.42 (1.47)	.036
Increase fiber consumption						
All participants	1.97 (2.44)	3.60 (2.66)	.002	1.86 (2.74)	3.31 (2.99)	.007
Subset with intentions to improve behavior at both assessments^b^	4.50 (1.22)	5.14 (1.10)	.045	5.18 (1.83)	5.72 (1.74)	.167
Increase physical activity						
All participants	4.51 (2.47)	4.80 (2.35)	.451	3.58 (2.85)	4.28 (2.60)	.149
Subset with intentions to improve behavior at both assessments^c^	5.33 (1.73)	5.74 (1.23)	.046	5.48 (1.60)	5.62 (1.20)	.576

*Abbreviations*: *M* mean, *SD* standard deviation

^a^Phase 1: *N* = 23, Phase 2: *N* = 24; ^b^Phase 1: *N* = 14, Phase 2: *N* = 11; ^c^Phase 1: *N* = 27, Phase 2: *N* = 21

## Discussion

The Families SHARE workbook was developed using a four step process, including a literature review to provide a basis for the FHH based risk algorithms and engagement of genetics education and health literacy experts and key stakeholders to evaluate the workbook’s content.

The resulting workbook provides users with an aesthetically pleasing and informative FHH tool that was understandable and engaging to mothers, a potential target group for FHH health education efforts. Additionally, the provided materials increased participants’ confidence in modifying their dietary behaviors over the short term, but not physical activity; however, confidence to increase physical activity was relatively high at initial assessment. Thus, the Families SHARE workbook holds promise in shifting key cognitive factors, such as intentions and confidence, associated with behavior change [25–27].

Many publically available tools offer a template for recording an individual’s FHH [9, 103, 104] or electronically generating risk assessments [11, 13]. The Families SHARE workbook does not directly provide a risk assessment; rather, it teaches users to recognize the pattern of disease in the family that increases their risk. Thus, the innovation is in the provision of a risk algorithm for users to assess whether they or their loved ones are at increased disease risk based on their FHH. All participants were able to use the algorithm to compute their own risk, and the majority was able to compute a family members’ risk. Research suggests that provision of a risk assessment is more motivating to sharing FHH with healthcare providers than provision of a pedigree only [7]. Future work will assess the effectiveness of the Families SHARE workbook in motivating sharing of FHH information within the family and with healthcare providers.

Recommendations for quantitative risk assessments were not integrated into the Families SHARE workbook. Accessibility of the workbook to those with limited literacy skills was an important factor in this decision [105]. Health literacy, including numeracy, is comprised of essential skills required for interpretation of health information – skills that have significant impact on health behavior and medical decision making [106–110]. Here the primary goal was to develop a plain language workbook that would be broadly accessible to the public. Numeracy can significantly impact understanding of quantitative risk assessments and risk perception. For this reason, the simplified algorithm that maps to “increased risk,” rather than a quantitative risk assessment, was retained. Moreover, messaging was based on an 8th grade reading level; all messaging, including the risk algorithm, was reviewed by experts in both health literacy and genetics education in an effort to design a tool that would have the potential to reach lower literacy populations. Disease risk assessment is complex, with many factors - genetic, behavioral, and environmental - contributing to risk. Admittedly, the algorithm provided in the workbook does not capture this complexity. Rather, the intent is to provide an educational tool that improves understanding of how FHH contributes towards risk – complex risk calculators may limit users ability to build mental models that link the pattern of disease within a family pedigree to disease risk.

One recommendation from study participants that warrants future investigation is to use the workbook to motivate behavior change through children’s risk. In response to Phase 1 feedback, worksheets were developed for computing risk for child and other family members and made available through the Families SHARE website; however, these worksheets were not accessed by Phase 2 participants. Only a third of participants collected FHH information from their child’s father, suggesting that the tool might need to be modified to more explicitly encourage this behavior if children are to be the target of health education efforts. The workbook can easily be modified to include both parents’ FHHs when personalizing the workbook pedigree and changing the language to focus behavioral recommendations towards children. The addition of developmentally appropriate screening recommendations stratified by risk, along with tips to motivate healthy lifestyles tailored to developmental stages would enhance such an application. Indeed, FHH may be successful in activating parental protection motivations targeted towards their children [111]. Recent research indicates that children’s risk due to FHH can influence parents’ child feeding behaviors [112]. Such results suggest that research aimed at motivating assessment and dissemination of risk information, as well as lifestyle modification within the home, might be more successful by focusing on children’s risk.

Follow-up interviews occurred, on average, two weeks following receipt of the workbook. It is encouraging that approximately a third of participants had talked with family members to gather FHH information after such a short period. However, this evaluation cannot speak to the long term impact of the tool. Several participants indicated that an electronic version of the pedigree and workbook would facilitate sharing of the information with family members. Indeed, online content can be easily updated, shared, monitored, and targeted; thus, an online FHH tool would be an ideal research infrastructure for capturing personal health behaviors and beliefs as well as social processes, such as sharing risk information. However, such an electronic version will not offer the “active learning” aspect of the simplified algorithm that is a fundamental part of the workbook. Future research will evaluate whether this active learning process is important to understanding of FHH and its impact on disease risk.

### Limitations

The Families SHARE workbook is a key step forward in understanding how to better make disease risk information accessible and actionable for families. However, the homogeneity of the stakeholder samples engaged in the development of the workbook potentially limits the usability of the workbook and generalizability of the findings to more diverse populations. The majority of participants had completed a bachelors or post-graduate degree and represented a higher income population. As previously discussed, reducing and simplifying the text throughout the workbook was a key revision to the workbook between Phase 1 and Phase 2, as well as defining technical terms, such as first- and second-degree relatives. However, the materials will need further evaluation if anticipating use in lower literacy populations, or with participants from under-resourced communities who may face greater barriers in adopting the recommended behaviors. Additionally, the workbook was evaluated by a primarily white sample; cultural context may be important, and thus the materials may need to be culturally tailored for other populations. Moreover, although research suggests that mothers may be optimally positioned for genomics health educator interventions [19, 20, 113], men and family members in older generations (e.g. grandparents) may also hold optimal positions in some families [14]. Thus, understanding and usability in these constituencies may warrant future investigation. Finally, the literature search informing the risk criteria and assessment language used in the workbook was relatively narrow, and as such may have missed relevant papers.

## Conclusion

The Families SHARE workbook was developed using a rigorous methodology. The final tool was found to be understandable and usable by key stakeholders. FHH is a genomic tool that is accessible across many populations. Knowledge of FHH has important implications in clinical care, informing health screening protocols. Additionally, understanding the role FHH plays in disease risk may have important implications in motivating engagement in risk reducing behaviors. The current report focused on the development and evaluation of the Families SHARE workbook. Ultimately, this tool will be used within a FHH-based intervention that engages mothers as genomics health educators with the goal of activating communal coping processes in an effort to improve family members’ health [23, 24].

## Additional file

Additional file 1: Table S1. Summary of qualitative risk language and associated risk criteria for colon cancer. **Table S2.** Summary of qualitative risk language and associated risk criteria for breast cancer. **Table S3.**Summary of qualitative risk language and associated risk criteria for heart disease. **Table S4. **Summary of qualitative risk language and associated risk criteria for type 2 diabetes. (DOCX 61 kb)

## Abbreviations

SHARE: Sharing Health Assessments and Risk Evaluations
FHH: Family health history
NHGRI: National Human Genome Research Institute
NIH: National Institutes of Health
AHRQ: Agency for Healthcare and Research Quality
FDR: First-degree relative
SDR: Second-degree relative
CRC: Colorectal cancer
BMI: Body mass index
CDC: Centers for Disease Control and Prevention
SPSS: Statistical Package for the Social Sciences
M: Mean
SD: Standard deviation
