# Supplementary material for: Evaluation of the Families SHARE workbook: an educational tool outlining disease risk and healthy guidelines to reduce risk of heart disease, diabetes, breast cancer and colorectal cancer
Source: BMC Public Health. 2015 Nov 13;15:1120. doi: 10.1186/s12889-015-2483-x (PMC4643512; doi:10.1186/s12889-015-2483-x)
Supplement: Additional file 1: Table S1. — Summary of qualitative risk language and associated risk criteria for colon cancer. Table S2. Summary of qualitative risk language and associated risk criteria for breast cancer. Table S3.Summary of qualitative risk language and associated risk criteria for heart disease. Table S4. Summary of qualitative risk language and associated risk criteria for type 2 diabetes. (DOCX 61 kb) [file 12889_2015_2483_MOESM1_ESM.docx]

Table S1: Colon cancer

| **Risk language** | **Risk criteria** |
| --- | --- |
| “approximately 40 percent increase in risk” [[1](#_ENREF_1)] | SDR or TDR with colon cancer |
| “high risk” [[1-5](#_ENREF_1)] | Affected FDR < age 50 |
|  | Affected FDR, late or unknown disease onset and affected SDR, premature disease from the same side of the pedigree |
|  | Affected FDR, premature disease onset |
|  | Affected FDRs (2+) |
|  | Affected relatives (3+) |
|  | Affected SDRs (2) with at least one having premature onset |
|  | Amsterdam criteria |
|  | Any combination of an affected FDR, 2 SDRs, or an SDR with premature disease onset |
|  | FDR with Lynch syndrome-related cancer ≤ age 50 years |
|  | FDR with polyp ≤ 50 years |
|  | HNPCC gene carriers and their FDRs |
|  | Relatives with HNPCC or FAP |
| “increased risk” [[1](#_ENREF_1), [2](#_ENREF_2), [6](#_ENREF_6)] | Affected FDR |
|  | Affected FDR ≥ age 50 |
|  | Affected FDR < age 50 |
|  | Affected FDRs (2) |
|  | Affected SDR < age 50 |
|  | FDR with advanced ademona(s) |
| “medium risk” [[4](#_ENREF_4)] | Affected FDRs (2) with 1 FDR < age 55 |
|  | Affected relative < age 45 |
|  | Affected relatives (2-3) with colorectal or endometrial cancer who are FDRs of each other, one diagnosed <age 55 and one a FDR of the consult and |
| “moderate risk” [[3](#_ENREF_3), [7](#_ENREF_7)] | Affected FDR ≥ age 60 with colorectal cancer or adenoma |
|  | Affected FDR ≤ age 59 with colorectal cancer or adenoma |
|  | Affected FDR, late or unknown disease onset |
|  | Affected FDRs (2) with colorectal cancer or adenoma |
|  | Affected SDRs (2) from the same lineage with late or unknown disease onset |

Table S2: Breast cancer

| **Risk language** | **Risk criteria** |
| --- | --- |
| “strong family history”[[8](#_ENREF_8)] | Affected FDRs (2+, female) with breast and/or ovarian cancer at any age  Affected FDR with breast cancer < age 50  Affected FDR with both breast and ovarian cancer at any age |
| “high risk” [[4](#_ENREF_4), [7](#_ENREF_7), [9-12](#_ENREF_9)] | Affected FDR |
|  | Affected FDR (mother or sister, postmenopausal) and an affected maternal SDR |
|  | Affected FDR (mother or sister, premenopausal) < age 50 |
|  | Affected FDR < age 50 |
|  | Affected FDRs (2+, female) with breast and/or ovarian cancer |
|  | Affected FDRs or SDRs (2+) on the same side of the family < 50 |
|  | Affected FDRs or SDRs with breast and ovarian cancer |
|  | Affected relative  Affected relative (2+) age 30-30 with breast cancer  Affected relative (3+) age 40-50 with breast cancer |
|  | Affected relatives (3+) < age 70 with breast or ovarian cancer |
|  | Affected relatives (4+) with breast cancer, ovarian cancer, or male breast cancer spanning 3 generations |
|  | Affected relatives (bilateral or multifocal) |
|  | Affected relatives (female) with breast and/or ovarian cancer |
|  | Affected SDR (premenopausal, maternal) and at least one other affected relative |
|  | Affected SDR (premenopausal, paternal) |
|  | Affected SDR via intervening male relative |
|  | BRCA1, BRCA2 or other predisposing gene |
|  | Early onset (undefined) |
|  | FDR of BRCA gene carrier |
| “potentially increased risk”[[13](#_ENREF_13)] | Affected relative (1+) with breast or ovarian cancer |
| “increased-risk family history” [[14](#_ENREF_14)] | Affected FDR (bilateral or multifocal) |
|  | Affected FDR or SDR with breast and ovarian cancer |
|  | Affected FDRs (2) with 1 FDR < age 50 |
|  | Affected FDRs or SDRs (3+) |
|  | Affected FDRs or SDRs with breast and ovarian cancer |
|  | Affected relative (male) |
|  | Ashkenazi Jewish woman with affected FDR (or 2 SDRs on the same side of the family) with breast or ovarian cancer |
|  | FDRs or SDRs (2+) with ovarian cancer |
| “Insignificant family history” [[15](#_ENREF_15)] | Absence of ≥ 2 relatives in a single bloodline with breast and/or ovarian cancer, or a relative with bilateral breast cancer or breast plus ovarian cancer |
|  | Affected FDR or SDR > age 40 with breast and/or ovarian cancer |
| “medium risk” [[4](#_ENREF_4)] | Affected FDR (bilateral) |
|  | Affected FDR (male) |
|  | Affected FDR < age 40 |
|  | Affected FDR and SDR < age 60 |
|  | Affected FDRs < age 60 |
|  | Affected FDRs or SDRs (3) on the same side of the family |
| “moderate risk” [[7-9](#_ENREF_7), [11](#_ENREF_11), [16](#_ENREF_16)] | Affected FDR with breast cancer > age 50  Affected FDR with ovarian cancer, but not breast cancer, at any age  Affected FDR or SDR < age 50 |
|  | Affected FDR or SDR < age 50 (male) |
|  | Affected FDR or SDR < age 50 with bilateral breast cancer |
|  | Affected FDR with late or unknown disease onset |
|  | Affected relatives (1-2) without nonbreast tumours  Affected relatives (2+) age 40-49 with breast cancer  Affected relatives (3+) age 50-60 with breast cancer |
|  | Affected relatives (3+) on one side of the family with breast or ovarian cancer |
|  | Affected SDRs (2) from the same lineage with late or unknown disease onset |
| “Modest family history” [[17](#_ENREF_17)] | Affected close relative > age 40 |
|  | Close relative with ovarian cancer and no FH of risk |
| “Insignificant family history” [[15](#_ENREF_15)] | Absence of ≥ 2 relatives in a single bloodline with breast and/or ovarian cancer, or a relative with bilateral breast cancer or breast plus ovarian cancer |
|  | Affected FDR or SDR > age 40 with breast and/or ovarian cancer |
| “personal and family history features suggestive of HBOC” [[18](#_ENREF_18)] | Affected relative (male)  Affected relative < age 40 |
|  | Affected relative with breast and ovarian cancer |
|  | Affected relatives (bilateral or multifocal) |
|  | Ashkenazi Jewish heritage and FH of breast cancer |
|  | Relative (female) with ovarian cancer and FH of breast or ovarian cancer |
| “Significant potentially high-risk family history” [[15](#_ENREF_15)] | Affected FDR or SDR < age 40 with breast and/or ovarian cancer |
|  | Affected relative with bilateral breast cancer or breast and ovarian cancer |
|  | Affected relatives (2+) in a single bloodline with breast and/or ovarian cancer |
| “slightly to moderately increased risk” [[10](#_ENREF_10)] | Affected FDR also having premenopausal ovarian cancer |
|  | Affected SDR |
|  | Average five-year Gail Model score with a first-degree relative post-menopause (older than 50 years) |
|  | Average five-year Gail Model score with a history of atypical ductal hyperplasia |
| “suggestive of hereditary breast/ovarian cancer syndrome that warrants further personalized risk assessment, genetic counseling, and often genetic testing and management” [[19](#_ENREF_19)] | Affected FDR or SDR ≤ 45   \| Affected FDR or SDR ≤ age 50 years with 1+ close blood relative* with breast cancer at any age or with a limited family history* \| \| --- \| \| Affected FDR or SDR and close male blood relative with breast cancer \| |
|  |  |
|  | Ashkenazi Jewish heritage or other ethnicity associated with higher mutation frequency |
|  | Close blood relative* (1+) with breast cancer diagnosed ≤ age 50 years |
|  | Close blood relative* (1+) with epithelial ovarian cancer |
|  | Close blood relatives* (2+) with breast cancer |
|  | Close blood relatives* (2+) with pancreatic cancer or aggressive prostate cancer (Gleason score ≥ 7) |
|  | FDR or SDR with epithelial ovarian cancer |
|  | FDR or SDR with male breast cancer |
|  | FDR or SDR with pancreatic cancer or aggressive prostate cancer (Gleason score ≥ 7) at any age with 2+ blood relatives with breast and/or ovarian and/or pancreatic or aggressive prostate cancer (Gleason score ≥ 7) at any age |
|  | Relative with a known deleterious BRCA1/BRCA2 mutation |
|  | TDR with breast and/or ovarian cancer with 2+ close relatives with breast cancer (at least one with breast cancer ≤ age 50 years) and/or ovarian cancer |
| “Suspected or known hereditary risk among women who have had breast cancer” [[17](#_ENREF_17)] | Affected close relative < age 50  Close relative with ovarian cancer |
| “Suspected or known hereditary risk among women who have not had breast cancer” [[17](#_ENREF_17)] | Affected close relative (male) |
|  | Affected close relatives (2) < age 40 |
|  | Affected close relatives (2) < age 50 and a second primary breast cancer at any age |
|  | Affected close relatives (2) and a third close relative with breast cancer , age 50, ovarian cancer, or male breast cancer |
| “Indications that hereditary susceptibility may be increased” [[20](#_ENREF_20)] | Affected relatives across multiple generations  Affected relatives with early onset |
|  | Multiple primary cancers |
|  | Relative with cancers in known syndrome |
|  | Relative with rare cancers |
|  | Relatives with ≥ 3 site-specific cancers |

Table S3: Heart Disease

| **Risk language** | **Risk criteria** |
| --- | --- |
| “high risk” [[7](#_ENREF_7)] | Affected FDRs (2+) |
|  | Affected relatives (3+) |
|  | Affected SDRs (2) with at least one having premature onset |
|  | Any combination of an affected FDR, 2 SDRs, or affected SDR with premature onset |
|  | FDR with late or unknown onset of disease and an affected SDR with premature disease on the same side of the pedigree |
|  | FDR with premature onset of disease |
| “increased the risk of developing CVD by about 1.3” [[21](#_ENREF_21)] | Affected FDR < age 55 (male)  Affected FDR < age 65 (female) |
|  |  |
| “moderate risk” [[7](#_ENREF_7)] | Affected SDRs (2) from the same lineage with late or unknown disease onset |
|  | FDR with late or unknown disease onset |
| Risk category: Average [[22](#_ENREF_22)] | Affected SDR |
| Risk category: Moderately high / Approximately 1.5 times increase in absolute risk score[[22](#_ENREF_22)] | Affected FDR |
|  | Affected SDRs (2) with colorectal cancer or adenoma |
| Risk category: Strong / Approximately two times increase in absolute risk score [[22](#_ENREF_22)] | Affected FDR and SDRs (2) |
|  | Affected FDRs (2) |

Supplementary Table 4: Diabetes

| **Risk language** | **Risk criteria** |
| --- | --- |
| “high familial risk” [[23](#_ENREF_23)] | Affected FDR and SDRs (2) |
|  | Affected relatives (2+) |
| “high risk” [[7](#_ENREF_7)] | Affected FDR with late or unknown onset of disease and an affected SDR with premature disease from the same side of the pedigree |
|  | Affected FDR with premature onset of disease |
|  | Affected FDRs (2+) |
|  | Affected relatives (3+) |
|  | Affected SDRs (2) with at least one having premature onset |
|  | Any combination of an affected FDR, 2 SDRs, or an affected SDR with premature onset of disease |
| “moderate familial risk” [[23](#_ENREF_23)] | Affected FDR |
|  | Affected SDRs (2+) |
| “moderate risk” [[7](#_ENREF_7)] | Affected FDR with late or unknown disease onset |
|  | Affected SDRs (2) from the same lineage with late or unknown disease onset |

**References**

1. Slattery ML, Kerber RA: **Family history of cancer and colon cancer risk: the Utah Population Database**. *J Natl Cancer* 1994, **86**(21):1618-1626.

2. Butterly LF, Goodrich M, Onega T, Greene MA, Srivastava A, Burt R, Dietrich A: **Improving the quality of colorectal cancer screening: assessment of familial risk**. *Dig Dis Sci* 2010, **55**(3):754-760.

3. Controlled Risk Insurance Company Risk Management Forum (CRIO/RMF): **CRICO/RMF Coloretal cancer screening algorithm: A decision support tool**. 2010.

4. National Services Scotland: **National data definitions for the national minimum core data set for cancer genetics**. 2006(Janurary).

5. Kastrinos F, Allen JI, Stockwell DH, Stoffel EM, Cook EF, Mutinga ML, Balmaña J, Syngal S: **Development and validation of a colon cancer risk assessment tool for patients undergoing colonoscopy**. *Am J Gastroenterol* 2009, **104**(6):1508-1518.

6. Network NCC: **Coloretal cancer screening**. *NCCN Guidelines®* 2013, **2.201**(Jan-98).

7. Scheuner MT, Wang SJ, Raffel LJ, Larabell SK, Rotter JI: **Family history: a comprehensive genetic risk assessment method for the chronic conditions of adulthood**. *American journal of medical genetics* 1997, **71**(3):315-324.

8. Halapy E, Chiarelli AM, Klar N, Knight JA: **Accuracy of breast screening among women with and without a family history of breast and/or ovarian cancer**. *Breast Cancer Research and Treatment* 2005, **90**:299 - 305.

9. Hoskins KF, Stopfer JE, Calzone KA, Merajver SD, Rebbeck TR, Garber JE, Weber BL: **Assessment and counseling for women with a family history of breast cancer. A guide for clinicians**. *JAMA* 1995, **273**(7):577-585.

10. Snyder LA, Soballe DB, Lahl LL, Nehrebecky ME, Soballe PW, Klein PM: **Development of the breast cancer education and risk assessment program**. *Oncol Nurs Forum* 2003, **30**(5):803-808.

11. Warner E, Goel V, Ondrusek N, Thiel EC, Chart PL, Meschino WS, Doan BD, Carroll JC, Taylor KM: **Pilot study of an information aid for women with a family history of breast cancer**. *Helath Expect* 1999, **2**(2):118-128.

12. Smith RP, Ni X, Muram D: **Breast cancer risk assessment: positive predictive value of family history as a predictor of risk**. *Menopause* 2011, **18**(6):621 - 624.

13. Hoskins KF, Zwaagstra A, Ranz M: **Validation of a tool for identifying women at high risk for hereditary breast cancer in population-based screening**. *Cancer* 2006, **107**(8):1769 - 1776.

14. U.S Preventive Service Task Force: **Genetic risk assessment and BRCA mutation testing for breast and ovarian cancer susceptibility: recommendation statement**. *Ann Intern Med* 2005, **143**(5):355-361.

15. Hughes KS, Roche C, Campbell CT, Siegel N, Salisbury L, Chekos A, Katz MS, Edell E: **Prevalence of family history of breast and ovarian cancer in a single primary care practice using a self-administered questionnaire**. *Breast J* 2003, **9**(1):19-25.

16. Eccles DM, Evans DGR, Mackay J: **Guidelines for a genetic risk based approach to advising women with a family history of breast cancer**. *Journal of Medical Genetics* 2000, **37**:203 - 209.

17. MacDonald DJ, Sarna L, Uman GC, Grant M, Weitzel JN: **Cancer screening and risk-reducing behaviors of women seeking genetic cancer risk assessment for breast and ovarian cancers**. In: *Oncol Nurs Forum: 2006*: Onc Nurs Society; 2006: E27-E35.

18. Korde LA, Gadalla SM: **Cancer risk assessment for the primary care physician**. *Prim Care* 2009, **36**(3):471-488.

19. Network NCC: **Gentic/familial high-risk assessment: Breast and ovarian**. *NCCN Guidelines®* 2013, **4.201**(Jan-76).

20. Kelly PT: **Hereditary breast cancer: risk assessment is the easy part**. *Breast J* 1999, **5**(1):52-58.

21. British Cardiac Society, British Hypertension Society, Diabetes UK HU, Primary Cardiovascular Society, and The Stroke Association: **JBS 2: Joint British Societies' guidelines on prevention of cardiovascular disease in clinical practice**. *Heart* 2005, **91 Suppl 5**:v1-52.

22. Hall R, Saukko PM, Evans PH, Qureshi N, Humphries SE: **Assessing family history of heart disease in primary care consultations: a qualitative study**. *J Fam Pract* 2007, **24**(5):435-442.

23. Valdez R: **Detecting undiagnosed type 2 diabetes: family history as a risk factor and screening tool**. *J Diabetes Sci Technol* 2009, **3**(4):722-726.
